# Supplementary figures and images for: The effects of gamelike features and test location on cognitive test performance and participant enjoyment
Source: PeerJ. 2016 Jul 6;4:e2184. doi: 10.7717/peerj.2184 (PMC4941792; doi:10.7717/peerj.2184)

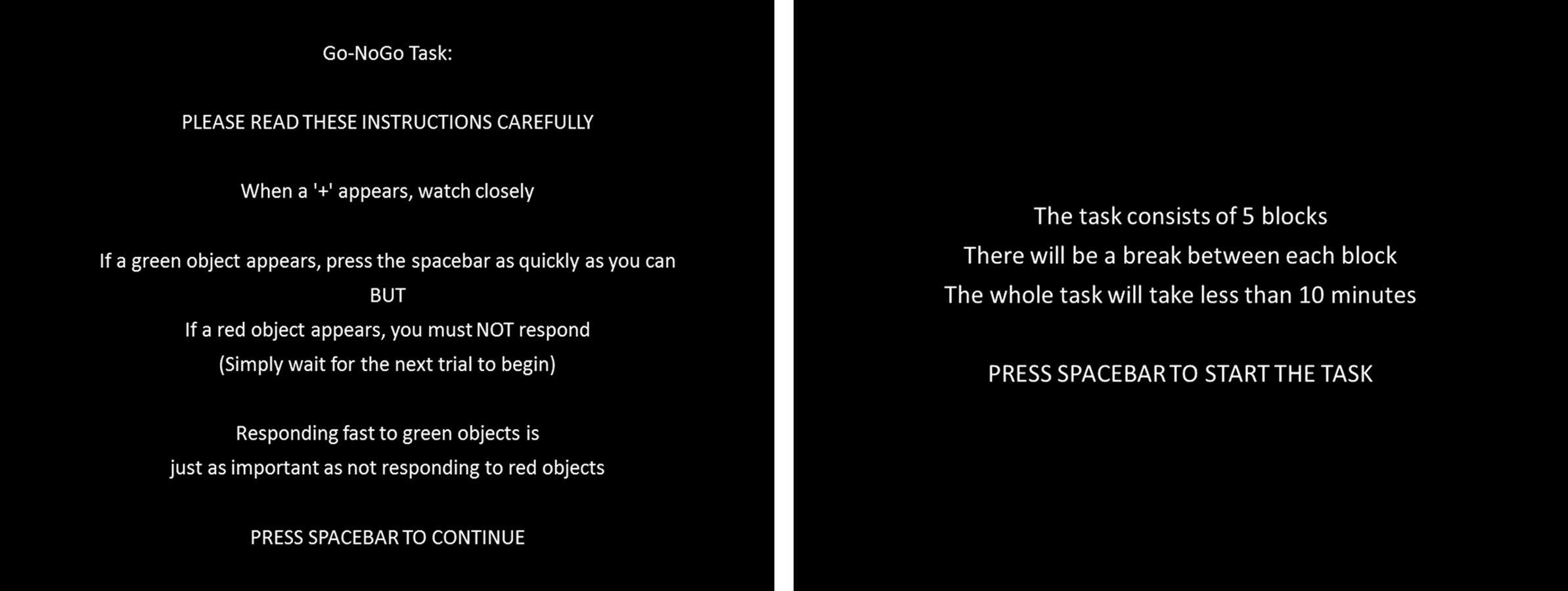

Supplement: Figure S1 [file peerj-04-2184-s001.png]

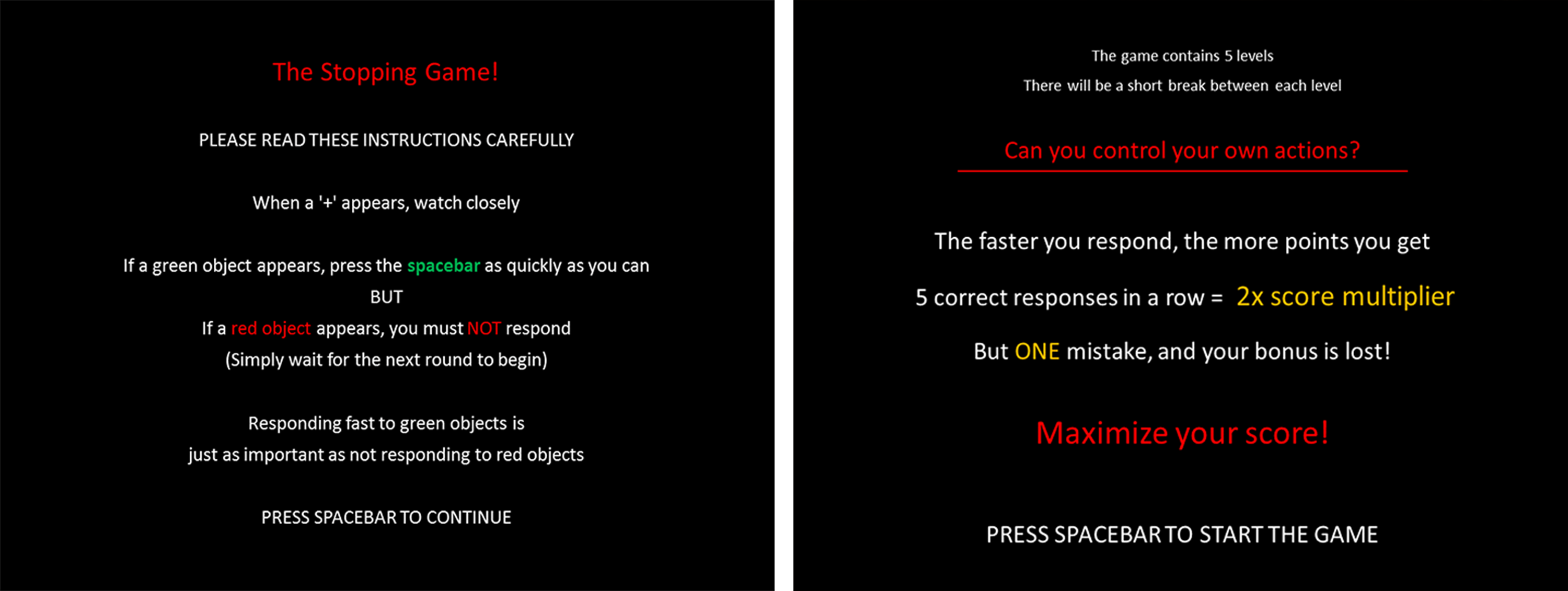

Supplement: Figure S2 [file peerj-04-2184-s002.png]

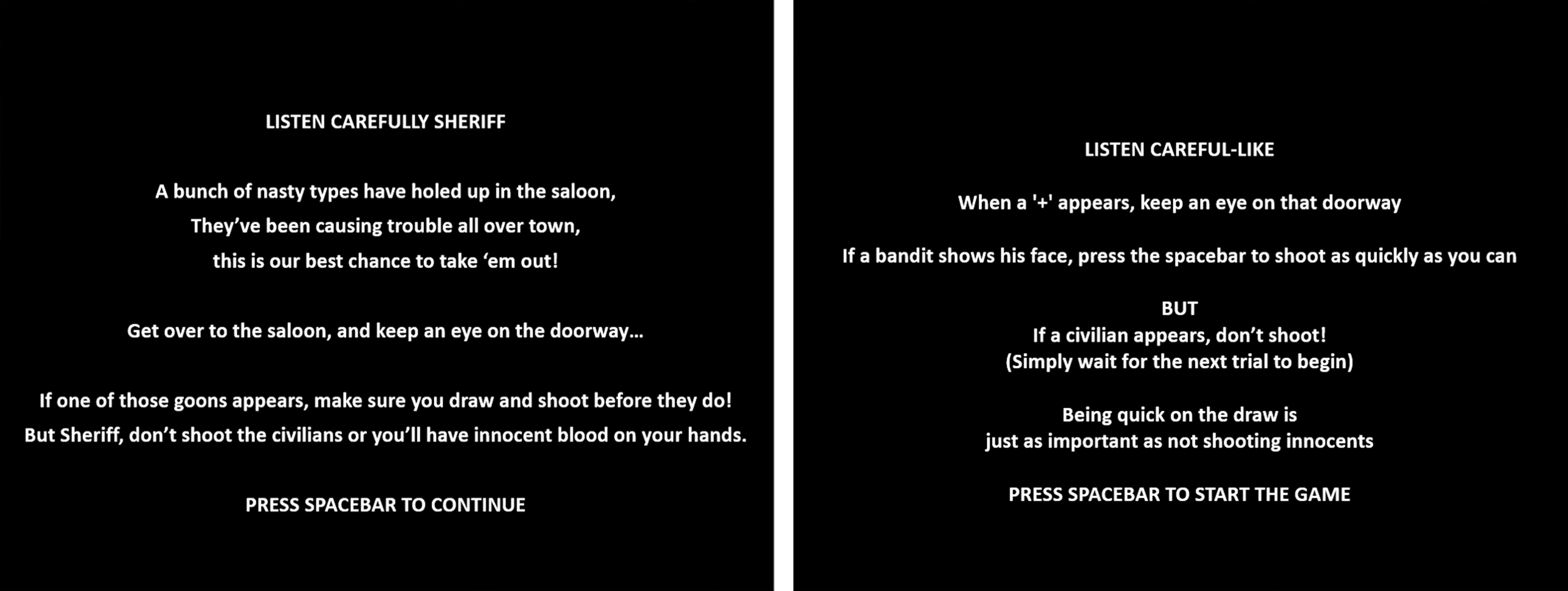

Supplement: Figure S3 [file peerj-04-2184-s003.png]
