# Supplementary material for: The effects of gamelike features and test location on cognitive test performance and participant enjoyment
Source: PeerJ. 2016 Jul 6;4:e2184. doi: 10.7717/peerj.2184 (PMC4941792; doi:10.7717/peerj.2184)
Supplement: Table S1 [file peerj-04-2184-s004.docx]

| Site | Variant | N | Median RT (95% CI) | Accuracy (95% CI) |
| --- | --- | --- | --- | --- |
| Online | Non-game | 67 | 401ms (392 - 410) | 97% (96.2 - 97.8) |
|  | Points | 71 | 402ms (393 - 412) | 98% (97.5 - 98.6) |
|  | Theme | 65 | 481ms (473 - 490) | 88.3% (86.1 - 90.5) |
| Lab | Non-game | 28 | 376ms (366 - 387) | 99.4% (99.1 - 99.7) |
|  | Points | 28 | 377ms (365 - 388) | 99% (98.3 - 99.7) |
|  | Theme | 28 | 469ms (459 - 480) | 92.3% (90.6 - 94) |
