# Supplementary material for: The effects of gamelike features and test location on cognitive test performance and participant enjoyment
Source: PeerJ. 2016 Jul 6;4:e2184. doi: 10.7717/peerj.2184 (PMC4941792; doi:10.7717/peerj.2184)
Supplement: Table S2 [file peerj-04-2184-s005.docx]

| Site | Variant | N | Median RT (95% CI) | Accuracy (95% CI) |
| --- | --- | --- | --- | --- |
| Online | Non-game | 67 | 368ms (350 - 385) | 93.8% (92.5 - 95.1) |
|  | Points | 71 | 372ms (355 - 389) | 95.1% (94.2 - 96) |
|  | Theme | 65 | 467ms (457 - 477) | 65.7% (61.7 - 69.6) |
| Lab | Non-game | 26 | 356ms (336 - 376) | 93% (90.7 - 95.3) |
|  | Points | 27 | 351ms (331 - 370) | 93.1% (91.1 - 95.2) |
|  | Theme | 28 | 448ms (436 - 461) | 65.6% (61.1 - 70.2) |
