# Supplementary material for: The effects of gamelike features and test location on cognitive test performance and participant enjoyment
Source: PeerJ. 2016 Jul 6;4:e2184. doi: 10.7717/peerj.2184 (PMC4941792; doi:10.7717/peerj.2184)
Supplement: Table S3 — Mann-Whitney U tests confirming the results of post-hoc t-tests on the non-normal accuracy data. m [file peerj-04-2184-s006.docx]

| Variable | Variant (N) | Variant (N) | U | Z-score | p-value | *r* |
| --- | --- | --- | --- | --- | --- | --- |
| Go Accuracy | Theme (93) | Points (99) | 648 | 10.27 | < .001 | 0.74 |
| Go Accuracy | Theme (93) | Non-game (95) | 892.5 | 9.45 | < .001 | 0.69 |
| Go Accuracy | Points (99) | Non-game (95) | 4277.5 | 1.09 | .275 | 0.08 |
| NoGo Accuracy | Theme (93) | Points (99) | 169.5 | 11.52 | < .001 | 0.83 |
| NoGo Accuracy | Theme (93) | Non-game (95) | 219 | 11.25 | < .001 | 0.82 |
| NoGo Accuracy | Points (99) | Non-game (95) | 4544 | 0.40 | .689 | 0.03 |
