# Supplementary material for: The effects of gamelike features and test location on cognitive test performance and participant enjoyment
Source: PeerJ. 2016 Jul 6;4:e2184. doi: 10.7717/peerj.2184 (PMC4941792; doi:10.7717/peerj.2184)
Supplement: Table S4 [file peerj-04-2184-s007.docx]

| Questions | Lab | | | Online | | |
| --- | --- | --- | --- | --- | --- | --- |
|  | Non-game | Points | Theme | Non-game | Points | Theme |
| *1) How enjoyable did you find the task?* | 45.0 (36.3 to 53.8) | 57.0 (47.1 to 66.9) | 52.6 (44.1 to 61.1) | 50.1 (44.1 to 56.1) | 59.2 (53.5 to 64.9) | 63.3 (56.6 to 69.9) |
| *2) How frustrating did you find the task?* | 29.5 (20.8 to 38.2) | 35.4 (25.9 to 45) | 40.8 (30.9 to 50.6) | 31.1 (24.4 to 37.7) | 38.4 (31.9 to 44.8) | 48.0 (41.2 to 54.8) |
| *3) How difficult was it to concentrate for the duration of the task?* | 40.3 (30.6 to 50) | 44.1 (33.2 to 55) | 39.0 (29.5 to 48.4) | 36.1 (29.4 to 42.8) | 37.4 (30.8 to 43.9) | 38.2 (30.7 to 45.6) |
| *4) How well do you think you performed on this task?* | 63.7 (56 to 71.3) | 60.7 (51.8 to 69.5) | 40.6 (32.6 to 48.6) | 72.1 (67.5 to 76.7) | 72.1 (68.1 to 76.1) | 51.5 (45.7 to 57.3) |
| *5) How mentally stimulating did you find this task to be?* | 35.8 (27 to 44.5) | 48.7 (39.1 to 58.2) | 50.9 (41.1 to 60.8) | 38.1 (31.7 to 44.6) | 56.2 (50.2 to 62.2) | 58.3 (51.2 to 65.4) |
| *6) How boring did you find the task?* | 61.4 (53.1 to 69.6) | 43 (31.9 to 54.2) | 41.4 (32.1 to 50.6) | 63.8 (57.5 to 70.2) | 44.1 (37.5 to 50.8) | 40.0 (32.9 to 47.2) |
| *7) How much effort did you put in throughout the task?* | 51.3 (39.4 to 63.1) | 71.3 (65.2 to 77.4) | 61.8 (53 to 70.7) | 83.1 (77.6 to 88.6) | 88.9 (84.9 to 92.8) | 89.9 (86.4 to 93.4) |
| *8) How repetitive was the task?* | 83.6 (77.1 to 90.1) | 79.3 (69.3 to 89.2) | 73.6 (65.7 to 81.6) | 89 (86 to 91.9) | 85.5 (81.7 to 89.4) | 80.3 (74.8 to 85.8) |
| *9) How willing would you be to take part in the study again?* | 68.3 (59.7 to 76.8) | 74.9 (67.7 to 82) | 68.7 (60.1 to 77.3) | 82.9 (78.1 to 87.8) | 82.2 (77.3 to 87.1) | 78.0 (71 to 85) |
| *10) How willing would you be recommend the study to a friend?* | 65.5 (55.9 to 75.1) | 78.1 (71.9 to 84.4) | 71.9 (64 to 79.8) | 70.7 (65.1 to 76.3) | 75.1 (69.9 to 80.3) | 71.1 (64.3 to 77.9) |
| *11) How intuitive did you find the pictures chosen for stop and for Go?* | 61.9 (56.4 to 67.4) | 63.4 (53.6 to 73.3) | 64.8 (58.2 to 71.3) | 64.5 (58.4 to 70.5) | 64.6 (59.1 to 70.1) | 63.9 (59.2 to 68.6) |
