# Supplementary material for: The effects of gamelike features and test location on cognitive test performance and participant enjoyment
Source: PeerJ. 2016 Jul 6;4:e2184. doi: 10.7717/peerj.2184 (PMC4941792; doi:10.7717/peerj.2184)
Supplement: Supplemental Information 1 [file peerj-04-2184-s008.docx]

Supplementary Analyses

The analyses below are included due to their mention as planned exploratory analyses in our preregistration on the Open Science Framework (<https://osf.io/b7eak/>).

### Exploratory Analysis 1: Do reaction times get longer as the test goes on? Does this effect differ between task variants?

We calculated a standardised Go RT for each trial based on the participant’s mean RT and standard deviation of their RT. We then averaged these standardised RTs within sub-blocks (12 trials per sub-block, 300 trials total) and over sites.

A two-way ANOVA of standardised Go RT gave clear evidence of a main effect of sub-block number (F [24, 75] = 14.547, *p* < .001, η² = .82), but not of task variant or an interaction (*p*s > 0.057). We used simple linear regression to assess the relationship between sub-block number and standardised Go RT in each group, and found good evidence for a relationship in each case (non-game: R^2^ = .44, F[1, 48] = 37.742, *p* < .001; points: R^2^ = .28, F[1, 48] = 18.752, *p* < .001; theme: R^2^ = .58, F[1, 48] = 65.578, *p* < .001). We plotted the data and regression lines, see Figure 1. We also calculated the modelled change in standardised Go RT from the first sub-block to the final sub-block, see Table 1.


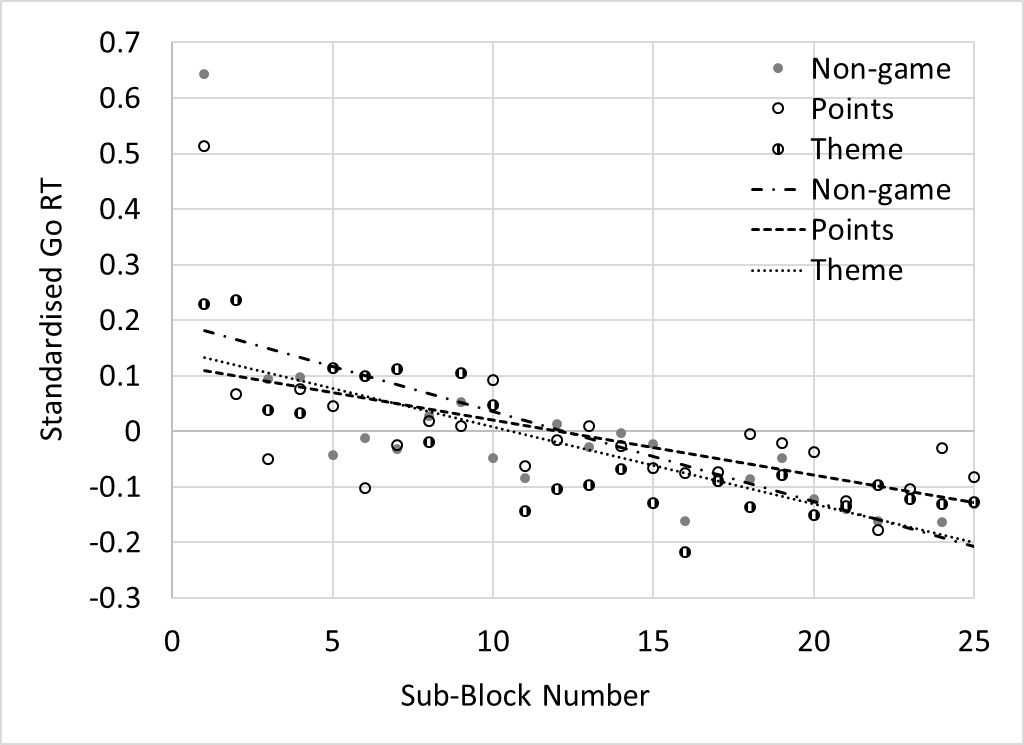


**Figure 1:** Standardised Go RT plotted against sub-block number, showing a downward trend as the experiment progresses.

| **Task Variant** | **Gradient** | **Intercept** | **Change in modelled standardised Go RT over whole task** |
| --- | --- | --- | --- |
| Non-game | -0.016 | 0.1982 | -0.39 |
| Points | -0.01 | 0.1193 | -0.24 |
| Theme | -0.014 | 0.1476 | -0.33 |

**Table 1:** Regression equations for each task variant, linking sub-block number to standardised Go RT.

Exploratory Analysis 2: Does No-Go accuracy get lower as the test goes on? Does this effect differ between **task variants**?

We calculated mean No-Go accuracy in each sub-block (3 No-Go trials in each), split by task variant. A two-way ANOVA of No-Go accuracy gave clear evidence of a main effect of Sub-block number (F [24, 75] = 3.807, *p* < .001, η² = .55) and task variant (F [2, 75] = 1463.407, *p* < .001, η² = .975). There was no evidence of an interaction (*p* = 0.071). Again, we used simple linear regression to assess the relationship between sub-block number and No-Go accuracy in each group, and found good evidence for a relationship in each case (non-game: R^2^ = .16, F[1, 48] = 9.361, *p* = .004; points: R^2^ = .25, F[1, 48] = 16.175, *p* < .001; theme: R^2^ = .33, F[1, 48] = 23.473, *p* < .001). We plotted the data and regression lines, see Figure 2, and calculated the modelled change in No-Go accuracy from the first sub-block to the final sub-block, see Table 2.


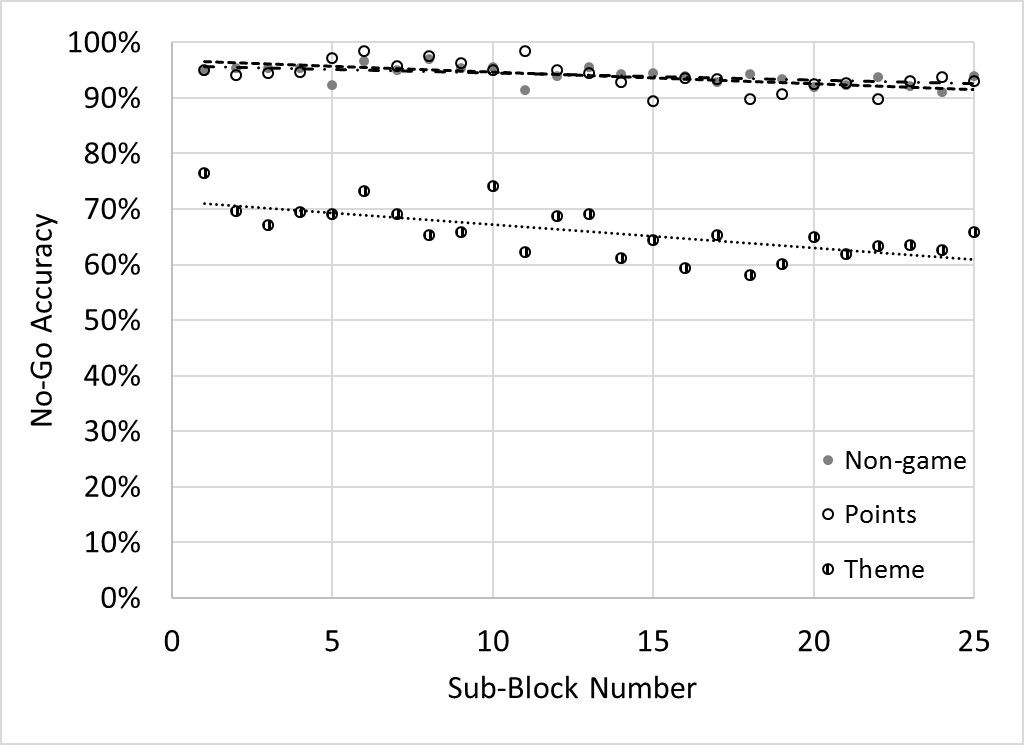


**Figure 2:** No-Go accuracy plotted against sub-block number

| **Task Variant** | **Gradient** | **Intercept** | **Change in modelled No-Go accuracy over whole task** |
| --- | --- | --- | --- |
| Non-game | -0.13% | 95.79% | 3.12% |
| Points | -0.21% | 96.72% | 5.04% |
| Theme | -0.42% | 71.50% | 10.08% |

**Table 2:** Regression equations for each task variant, linking Sub-block number to No-Go accuracy.

Discussion:

Contrary to expectations, RTs did not get longer as the task went on. We anticipated that boredom might cause participants to become inattentive and therefore respond more slowly, however we found strong evidence of an opposite effect, with RT speeding up over the course of the task. We also saw no impact of task variant, implying that this speed up was not related to engagement or enjoyment of the task. One explanation for the effect might be that the as the task progressed, the stimuli became more familiar and could be responded to faster, i.e. learning was occurring (Verbruggen & Logan, 2008). Another possible explanation is that participants grew bored in all task variants, and simply responded more rapidly in order to “get the task over with”.
 No-Go accuracy showed a similar pattern, with accuracy decreasing as the task progressed. However this was in line with our hypotheses, and it is possible that the decrease in No-Go accuracy was the result of participant boredom. We saw little evidence for an interaction between task variant and sub-block number, although the Theme variant did show the largest change in accuracy from the beginning to the end of the task; ruling out the idea that a more engaging task might alleviate the effect of boredom (if this was indeed a boredom effect). Accuracy change over time in the other two variants was quite small.
 An alternative explanation for the decrease in No-Go accuracy over time is that the loss of accuracy was caused by the decrease in RTs, i.e. a speed-accuracy trade-off (Bogacz, 2013; Wickelgren, 1977). We calculated three Pearson correlations between standardised Go RT and No-Go accuracy in each sub-block, split by task variant (non-game: *r*(50) = .301, *p* = .034 ; points: *r*(50) = .24, *p* = .093; theme: *r*(50) = .582, *p* < .001). These findings indicate that the relationship between RT and accuracy was strongest in the non-game and theme variants. Tentatively, one might hypothesise that the incentive structure facilitated by the points task prevented a speed-accuracy trade-off from occurring. However, replication of these findings would be required in order to draw any firm conclusions.

References:

Bogacz, D. R. (2013). Speed-Accuracy Trade-Off. In D. Jaeger & R. Jung (Eds.), *Encyclopedia of Computational Neuroscience* (pp. 1–4). Springer New York. Retrieved from http://link.springer.com/referenceworkentry/10.1007/978-1-4614-7320-6_319-1

Verbruggen, F., & Logan, G. D. (2008). Automatic and controlled response inhibition: Associative learning in the go/no-go and stop-signal paradigms. *Journal of Experimental Psychology: General*, *137*(4), 649–672. http://doi.org/10.1037/a0013170

Wickelgren, W. A. (1977). Speed-accuracy tradeoff and information processing dynamics. *Acta Psychologica*, *41*(1), 67–85. http://doi.org/10.1016/0001-6918(77)90012-9
